# Autistic disorder associated with a paternally derived unbalanced translocation leading to duplication of chromosome 15pter-q13.2: a case report

**DOI:** 10.1186/1755-8166-2-27

**Published:** 2009-12-18

**Authors:** David J Wu, Nicholas J Wang, Jennette Driscoll, Naghmeh Dorrani, Dahai Liu, Marian Sigman, N Carolyn Schanen

**Affiliations:** 1 Department of Biological Sciences, University of Delaware, Newark, USA; 2 Center for Applied Clinical Genomics and Center for Pediatric Research, Nemours Biomedical Research, Alfred I duPont Hospital for Children, Wilmington, Delaware, USA; 3 Department of Human Genetics, Geffen School of Medicine, University of California, Los Angeles, California, USA; 4 Division of Biotechnology, California South Bay University, Sunnyvale, California, USA; 5 Neuropsychiatric Institute, Geffen School of Medicine, University of California, Los Angeles, California, USA; 6 Department of Pediatrics, Thomas Jefferson University, Philadelphia, Pennsylvania, USA

## Abstract

Autism spectrum disorders have been associated with maternally derived duplications that involve the imprinted region on the proximal long arm of chromosome 15. Here we describe a boy with a chromosome 15 duplication arising from a 3:1 segregation error of a paternally derived translocation between chromosome 15q13.2 and chromosome 9q34.12, which led to trisomy of chromosome 15pter-q13.2 and 9q34.12-qter. Using array comparative genome hybridization, we localized the breakpoints on both chromosomes and sequence homology suggests that the translocation arose from non-allelic homologous recombination involving the low copy repeats on chromosome 15. The child manifests many characteristics of the maternally-derived duplication chromosome 15 phenotype including developmental delays with cognitive impairment, autism, hypotonia and facial dysmorphisms with nominal overlap of the most general symptoms found in duplications of chromosome 9q34. This case suggests that biallelically expressed genes on proximal 15q contribute to the idic(15) autism phenotype.

## Background

The association of maternally derived chromosome 15 duplications and autism spectrum disorders (ASD) has been well defined (reviewed in [1]). The classic duplications and deletions in this region involve non-allelic homologous recombination events between a group of low copy repeats (LCR) located on the proximal long arm that generate common breakpoints (BP) in most of the derivative chromosomes [2]. Because most duplications are maternally derived and the region is subject to genomic imprinting, much of the focus for the duplication phenotypes has been on the maternally expressed transcripts although it is likely that the full phenotype arises from contiguous gene effects, also involving the biallelically expressed genes included in the duplication chromosomes.

The phenotypic consequences of paternally derived duplications are not well delineated. Idic(15) chromosomes are almost exclusively maternally derived with the only known paternally derived idic(15) chromosomes occurring in mosaic form in association with maternal uniparental disomy and Prader Willi syndrome (PWS) [3,4]. Other paternally derived duplications of this region are interstitial rearrangements that were identified in familial cases in which a phenotypically normal mother transmitted a paternally derived duplication chromosome, leading to ASD in the child [5]. Rare cases of paternal duplications associated with developmental delays and possibly ASD have been reported [6,7]. Here we describe a case of an autistic boy who is trisomic for 15pter-q13.2 and 9q34.12-qter because of a segregation error leading to transmittance of two copies of a balanced paternal translocation chromosome derived from chromosomes 9 and 15. His behavioral profile overlaps the duplication 15q11.2-q13.2 phenotype including the presence of autism, suggesting that the maternally expressed genes on chromosome 15q are not solely responsible for the ASD phenotype. Notably, he is trisomic for distal 9q, however, neither deletions nor duplications of this region have been associated with autistic symptoms [8-11].

## Case Presentation

### Clinical History

The child was the product of the third pregnancy to a 34-year old mother and 36-year old father. The couple had previously had one first trimester spontaneous pregnancy loss. Reduced fetal movements were noted throughout the pregnancy. He was born by Cesarean section after failed induction of delivery at 42 weeks gestation. Birth weight was 2500 g (3-10%ile) and length 54.6 cm (25-50%ile); head circumference data were not available. Apgar scores were 8 and 8 at one and five minutes, respectively. In the immediate neonatal period, he was noted to be hypotonic, with poor suck and swallow, and had temperature instability. He failed attempts to breastfeed and required bottle-feeding with an adapted nipple. At one week of age an umbilical hernia was identified, which resolved spontaneously. He had minor dysmorphic facial features including bilateral epicanthal folds. His eyes were not deepset. He had a low nasal bridge without evidence of beaking but with mildly hypoplastic nasal alae, poorly defined philtrum with down turned corners of the mouth, a submucosal cleft palate and prominent, incompletely folded ears. Testes were descended bilaterally. Hand length and finger lengths were normal (50-75%ile) with no increases in digital creasing and normal thumb placement. He had pes planus and a valgus deformity of the ankles, which were managed with ankle-foot orthoses from 13 mos to age 6 years, with surgical repair at 14 years of age. At 12 mos of age, parental concerns regarding his development were raised and he was referred to neurology because of global developmental delays at 17 mos of age. Cranial magnetic resonance imaging and cerebral tomography were normal. High resolution chromosome analysis revealed an unbalanced translocation, designated by the karyotype, 47,XY,t(9;15)(q34.3;q13),+der(15)t(9;15)(q34.3;q13). The maternal karyotype was normal and the father was found to carry a balanced translocation between chromosomes 9 and 15, designated by the karyotype, 46,XY,t(9;15)(q34.3;q13). Medical history is significant for numerous episodes of otitis media with tympanostomy tubes placed in infancy, repeated sinus infections, and seasonal allergies. He underwent adenoidectomy at age 14 yrs. Puberty is delayed, with Tanner I-II changes at age 15 yr. An echocardiogram and electrocardiogram were normal.

He was enrolled in early intervention programs in infancy that included physical, occupational and speech therapy. Early development was notable for global delays, with pronounced deficits in social and language acquisition. The age at first word was 78 mos, with onset of phrase speech by 90 mos of age. Early speech was echolalic with problems with fluency, articulation errors, and hypernasality. Gross motor milestones were delayed. He sat with support at 9 mos and independently at 15 mos, and walked at 33 mos but required ankle foot orthoses and physical therapy, both of which continued until age 7.5 years. As a toddler, he continued to have problems feeding because of sensory aversions. He made poor eye contact, had frequent tantrums, cried excessively and had difficulties separating from parents. Disrupted sleep patterns have been present since infancy and persist to date.

He entered preschool at age 3 years where he was socially withdrawn and had poor attention without hyperactivity. He displayed aggressive behaviors including biting and hitting himself and others. He had difficulties transitioning to new activities and changes in routine. Psychological testing using the Bayley Scales of Infant development performed at age 37 mos showed gross and fine motor skills at the 24-36 mos level, cognition at 24 mos, language at 18 mos and social skills at the 12 mos age equivalent. Repeated testing at age 5 y 3 mos indicated an intelligence quotient of 86 on the Academic Scale of Development Profile II, with most pronounced deficits in social and language tasks.

Following informed consent and parental permission using protocols approved by the UCLA Human Subjects Protection Committee, formal neuropsychiatric testing was done at age 11 y 11 mos as part of our study of chromosome 15 duplications in autism. Using the Stanford-Binet Intelligence Scales, fourth edition [12], he achieved an age equivalent of 7 y 5 mos on the bead memory test, which assesses short-term memory and 4 y 6 mos on the pattern analysis subtest, which examined his ability to analyze and copy patterns shown to him. On the quantitative reasoning and vocabulary subtests, he achieved an age equivalents of 6 y 10 mos and 4 y 3 mos, respectively. Language skills were assessed using the CELF-R [13] as well as non-standardized assessment of non-verbal communication [14]. The CELF-R measures both receptive and expressive language and he achieved an age equivalent of less than 5 years in each domain. The non-verbal language assessment measured joint attention, requesting behaviors and social interactions. He demonstrated many of these behaviors, looking back and forth from the experimenter to active toys, and following the experimenter's points in a book and around the room. However, he did not engage in social interaction with the experimenter.

The VABS were used to measure adaptive behaviors [15]. Based on responses obtained in the parent interview, he received age equivalences of 93 mos in the Communication domain, 42 mos in Daily Living Skills, and 38 mos in Socialization. The Adaptive Behavior Composite takes into account each of these four subsections and he received an overall age equivalence of 58 mos. A diagnosis of autism was made using the ADI-R [16] and the ADOS-G Module 3 [17]. He met criteria for autism in the three domains assessed in the ADI-R, with scores of 25 for the social behavior, 16 for communication and 5 for restrictive and repetitive behaviors. He also met narrow criteria for autism on module 4 of the ADOS-G.

### Molecular and cytogenetic studies

**Figure 1:**
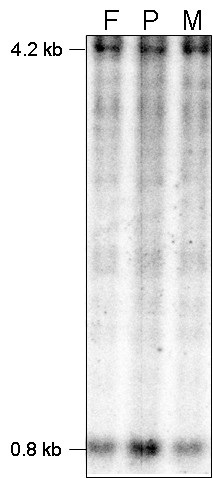
**Quantitative Southern Blot analysis of the SNRPN exon α locus to determine gene dosage and methylation status**. Southern blot analysis was performed using the SNRPN exon α probe, which detects a methylated maternal band at 4.2 kb and unmethylated paternal band at 0.8 kb. Densitometry was performed on the blot, which indicated that the proband (P) shows increased dosage of the paternal unmethylated allele when compared to the parental samples (F: Father, M: Mother) with a ratio of 1.47:1, consistent with paternal methylation of the der(15) chromosome. This was further supported by methylation specific PCR, which showed paternal:maternal ratio of 1.8:1 (not shown).

Genotyping was performed with a group of 29 short tandem repeat polymorphism (STRP) markers from chromosome 15, nine of which revealed evidence for an isodisomic paternal duplication of chromosome 15 based on increased peak heights for markers proximal to BP4 (Table 1). Similarly, genotyping was performed with 20 STRP markers from chromosome 9, which showed increased dosage at two of the most telomeric STRP markers based on peak heights (Table 2). Homozygosity for the parents and proband at STRP marker, D9S164, which is likely also included in the duplication may have precluded detection of a duplication at this locus because it was not possible to accurately compare peak height among the three individuals. Southern analysis of the SNRPN exon α locus showed a 1.47:1 dosage ratio of paternal to maternal alleles, consistent with paternal methylation of the derivative chromosome (Figure 1). Methylation specific PCR at this locus confirmed this result, with amplification of alleles at a ratio of 1.8:1 for unmethylated to methylated fragments (not shown).

**Table 1:** Chr. 15 genotyping shows paternal isodisomic inheritance.

STRP	Location (Mb)	Mother	Proband	Father	Position
D15S542	20.35	2,2	1,1,2	1,1	BP1-BP2
D15S817	22.06	1,2	2,2,2	1,2	BP2-BP3
D15S1021	22.47	1,2	1,1,1	1,2	BP2-BP3
GABRB3	24.34	1,2	1,3,3	3,3	BP2-BP3
D15S97	24.28	2,3	2,2,2	1,2	BP2-BP3
D15S822	24.87	2,3	3.4,4	1,4	BP2-BP3
D15S1019	27.35	2,2	2,2,2	1,2	BP3-BP4
D15S1048	27.56	3,3	1,1,3	1,2	BP3-BP4
D15S1043	27.93	1,1	1,2,2	2,3	BP3-BP4
D15S165	28.95	1,2	1,1	1,2	BP4-BP5
D15S184	29.18	1,2	1,2	2,2	BP4-BP5
D15S1013	29.42	1,2	1,3	2,3	BP4-BP5
D15S1010	30.78	1,2	1,2	2,2	DISTAL
D15S1007	31.42	1,3	1,2	1,2	DISTAL
D15S123	45.75	1,2	1,2	1,3	DISTAL

**Table 2:** Chr. 9 genotyping shows paternal isodisomic inheritance.

STRP	Location (Mb)	Mother	Proband	Father
D9S288	3,84	2,3	1,2	1,4
D9S286	7.94	1,2	1,2	1,2
D9S285	15.97	1,3	3,3	2,3
D9S157	17.52	2,4	1,2	1,3
D9S171	24.42	1,1	1,1	1,2
D9S161	27.52	2,3	1,3	1,2
D9S1817	33.75	1,3	1,2	1,2
D9S273	71.63	1,2	2,2	2,3
D9S175	77.04	1,4	2,4	2,3
D9S167	84.87	3,4	1,4	1,2
D9S283	91.50	3,4	2,4	1,2
D9S287	97.41	1,3	1,3	1,2
D9S1690	103.04	1,2	2,3	1,3
D9S1677	110.88	2,3	2,2	1,2
D9S1776	116.90	2,3	1,2	1,3
D9S1682	123.93	2,3	1,3	1,3
D9S290	130.47	1,2	2,2	2,2
D9S164	135.15	1,1	1,1	1,1
D9S1826	137.49	2,3	1,1,3	1,3
D9S158	138.14	1,3	1,2,2	2,4

**Figure 2:**
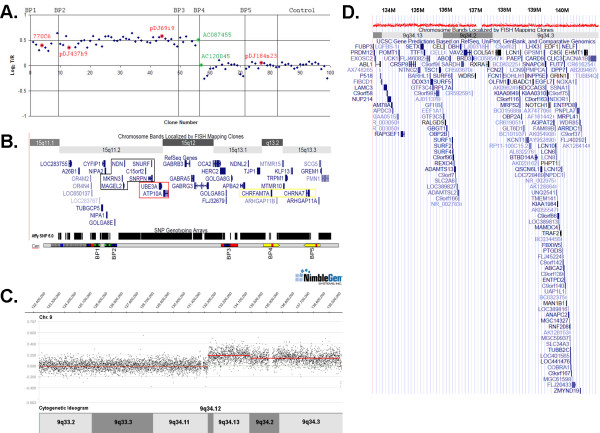
**Log_2 _T/R ratio plots for comparative genome hybridization**. A) Custom BAC Array CGH of chromosome 15 shows trisomy up to BP4. FISH probes used are circled in red. Notably, FISH probe pDJ204m06 was not used on the array thus is not shown. Clones used for BLAST search for homology against chromsome 9 BP are circled in green. B) Schematic of chromosome 15q11.1-13.3 showing the position of genes based on the UCSC genome browser. Highlighted in red are maternally expressed transcripts, and paternally expressed transcripts in black. (Below) The relative positions of the 5 BP clusters are shown with sequence homology indicated by color, blue indicating regions of homology to *HERC2*, green indicating regions of homology to *GOLGA8E*, yellow indicating regions of homology between *CHRNA7*. The black and white hatching indicates the heteromorphic region near the centromere that includes a number of pseudogenes and can expand in the normal population. Above the breakpoint schematic is the Affymetrix 6.0 whole genome array that shows the density of SNP coverage for this region with notable gaps at the positions of the common BPs, although not all probes for detecting copy number variations are shown in the UCSC browser. C) Nimble CGH Array of chromosome 9 shows trisomy distal to position 132,510,300 (Build 36.1) in 9q34.1. D) Duplicated genes in chromosome 9 based on the UCSC genome browser.

To further assess the dosage and BP position for the translocation event on chromosome 15, array CGH was performed using our custom BAC array, revealing dosage consistent with trisomy for the interval between BP1 and BP4 (clone AC087455) on chromosome 15q13.2 (Figure 2A and 2B) [18]. The position of the chromosome 9 BP was interrogated using array CGH with the Nimblegen platform. These analyses placed the proximal boundary of the translocation event on chromosome 9 at g 132,510,300 (Figure 2C and 2D). The 2 kb region flanking the translocation BP on chromosome 9 is composed of 52.6% short interspersed elements and 24.3% long interspersed elements. We performed a BLAST search comparing a 2 kb region flanking the translocation BP on chromosome 9 with the two clones flanking the BP on chromosome 15 as well as the intervening sequence and detected two primary blocks of homology. For BAC AC087455, a 283 bp sequence (g 28,082,283 - 28,082,562) with 87% identity to the chromosome 9 BP sequence (g 132,510,495-132,510,776) lies near the telomeric end of the BAC clone. Similarly, a 283 bp region with 90% identity was detected between clone AC120045 (g 28,136,197-28,136,479) and the chromosome 9 sequence. Either of these regions of sequence homology may have facilitated a non-homologous allelic recombination event that gave rise to the original translocation chromosome. Subsequently, a 3:1 segregation error in the paternal germline led to aneusomy for the der(15) in the proband.

**Figure 3:**
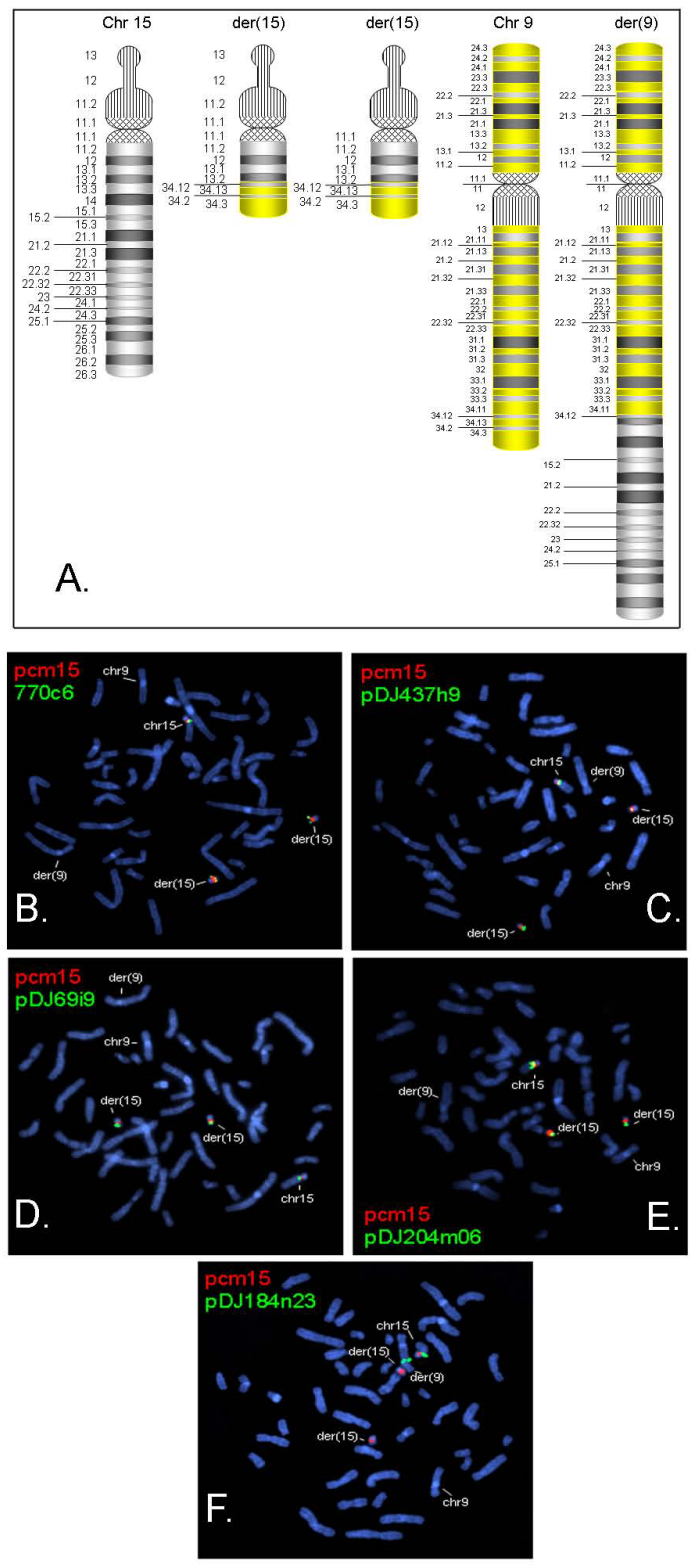
**Characterization of translocation chromosomes by metaphase FISH**. The two der(15) chromosomes revealed hybridization signals for clones up to BP4 and no signal distal to BP5. Der(9) chromosome revealed hybridization signal for pDJ184n23, which is distal to BP5. A) Schematic representation of chromosomes 9, 15 and the derivative chromosomes. In panels B-F, red signals represent pcm15, a chromosome 15 centromere probe. The positions of the other clones used for FISH are shown in Figure 2A. Chromosomes 9, 15 and the derivative chromosomes are indicated in each panel. B) Green signals represent clone 770c6, which lies in proximal to BP2. C) Green signals represent clone pDJ437h9, which lies in the BP2-BP3 interval. D) Green signals represent clone pDJ9i9, a BP2-BP3 probe that lies just proximal to BP3. E) Green signals represent clone pDJ204m06, which lies between BP3-BP4. F) Green signals represent clone pDJ184n23, which lies distal to BP5.

FISH was performed with cosmid clones pDJ77oC6, 437h9, pDJ69i9, pDJ204m06 and pDJ184n23 (Figure 3A-3E). These studies revealed single hybridization signals on each of the der(15) chromosomes for the probes proximal to BP4 and no signal on the der(15) for probe184n23, which lies distal to BP5 (Figure 3F). These studies confirmed the presence of a non-mosaic translocation chromosome that extended through pDJ204m06 at 15q13.2 (27.8 Mb).

## Conclusions

This is a unique case of a paternally derived duplication of the chromosome 15pter-q13.2 region that has been associated with ASD. His duplication arises from a 3:1 segregation error of a balanced translocation in the paternal germline. The translocation appears to have been mediated by a region of sequence homology between the LCR at BP4 on chromosome 15 and chromosome 9. The resolution of the array based approaches used to detect the position of the BPs on both chromosomes provided clues to the potential mechanism that contributed to the original recombination because it was possible to search for sequence homology at the BP sites. Because the BP on chromosome 15 falls in a complex LCR [19], we do not know which of the two positions is specifically involved in the exchange, nonetheless, it is likely that a non-allelic recombination event between related sequences led to the translocation on the father's chromosome. Methylation analyses indicate the duplication has retained its paternal imprint.

This boy presents with many of the clinical manifestations associated with the dup(15) syndrome phenotype including autism, hypotonia, epicanthal folds and learning disabilities [20]. Some of these features were also seen in a case of paternally derived interstitial duplication who manifest developmental delays including language impairments [7]. The other family that was reported with a paternally derived interstitial duplication had two affected probands however the duplication did not cosegregate with phenotype in the extended family, raising questions as to its pathogenicity [6]. Notably, the case reported here also displays some features of the duplication chromosome 9q34.1 syndrome including low birth weight, with normal birth length and valgus deformities at the ankles. Other aspects of the duplication 9q34 duplication syndrome are not present such as dolichocephaly, beaked nose, deepset eyes, microphthalmia and arachnodactyly [11,21]. While the genomic segment that is duplicated on chromosome 9 is gene rich and includes a number of genes that might be predicted to have developmental consequences, autism has not been previously reported associated with duplications of chromosome 9q34.1-qter [8,11,21] nor in deletions of the region [22,23]. Moreover, the region on chromosome 9 has not been linked to autism in genome wide screens for autism susceptibility genes, leading one to suspect that the autism component of his phenotype arises from the chromosome 15 duplication. The derivative chromosome 15 is paternally derived and appears to have maintained its paternal imprint, thus raising the possibility that biallellically expressed genes within the duplicated segment are important contributors for ASD arising from duplications of chromosome 15.

## Methods

### Molecular and cytogenetic studies

The subjects were enrolled following informed consent using a protocol approved by the Human Subjects' Protection Committee at the University of California, Los Angeles. Blood samples were obtained from the child and both parents for DNA extraction and generation of Epstein-Barr virus immortalized lymphoblast cell lines (LCL) using standard techniques. DNA was extracted from peripheral blood leukocytes and LCL using the Gentra Puregene DNA extraction kit (Qiagen, Valencia CA) per the manufacturer's instructions. Genotyping was performed using 29 short tandem repeat polymorphism (STRP) markers from chromosome 15q and 19 from chromosome 9 (Tables 1 and 2) [24]. Methylation analyses at exon alpha of the SNRPN locus was examined by Southern blot and densitometry [25] and methylation specific PCR [26].

Genomic DNA from LCL was used for array CGH as described in Wang et al. (2004) [18]. The position of the breakpoint on chromosome 9 determined by array CGH using LCL DNA hybridized to the chromosome 9 specific array (Nimblegen, Inc., Madison WI).

Phenotypic analyses of the proband were performed by examination of medical records, parent interview and by direct assessment in the subject's home using the Stanford Binet Intelligence Scales, fourth edition [12], Clinical Evaluation of Language Fundamentals-Revised (CELF-R) [13], Autism Diagnostic Interview- Revised (ADI-R) [16], Autism Diagnostic Observation Schedule-generic (ADOS-G) [17], and Vineland Adaptive Behavior Scales (VABS) [15].

## Consent

Consent was obtained from the family for publication at the time of enrollment into the study. A copy of the written consent is available for review by the Editor-in-Chief of this journal.

## Competing interests

The authors declare that they have no competing interests.

## Authors' contributions

DJW participated in the molecular and cytogenetic studies, performed the breakpoint analysis including the bioinformatics evaluation of the two segments of DNA involved in the rearrangements. He was also responsible for directly drafting the manuscript. NJW performed the chromosome 15 array CGH, SNRPN Southern Blot Analysis and dosage analysis of the duplication. JD assisted in the molecular and cytogenetic studies. ND was the study coordinator who assisted with recruitment of the family and ascertainment of clinical data. DL performed the genotyping and portions of the dosage analysis. MS oversaw collection of formal neuropsychiatric testing as part of the study protocol. NCS designed the study, coordinated collection of clinical data from parents and medical records and drafted the manuscript. All authors read and approved the final manuscript.

## References

[B1] Hogart A Wu D Lasalle JM Schanen NC The comorbidity of autism with the genomic disorders of chromosome 15q11.2-q13 Neurobiol Dis 2008 in press 18840528 10.1016/j.nbd.2008.08.011 PMC2884398

[B2] Pujana MNM Guitart M Armengol L Gratacos M Estivil X Human chromosome 15q11-q14 regions of rearrangements contain clusters of LCR15 duplicons European Journal of Human Genetics 2002 10 26 35 10.1038/sj.ejhg.5200760 11896453

[B3] Baumer A Wiedemann U Hergersberg M Schinzel A A novel MSP/DHPLC method for the investigation of the methylation status of imprinted genes enables the molecular detection of low cell mosaicisms Hum Mutat 2001 17 5 423 430 10.1002/humu.1118 11317358

[B4] Saitoh S Hosoki K Takano K Tonoki H Mosaic paternally derived inv dup(15) may partially rescue the Prader-Willi syndrome phenotype with uniparental disomy Clin Genet 2007 72 4 378 380 17850637 10.1111/j.1399-0004.2007.00860.x

[B5] Cook EH Jr Lindgren V Leventhal BL Courchesne R Lincoln A Shulman C Lord C Courchesne E Autism or atypical autism in maternally but not paternally derived proximal 15q duplication Am J Hum Genet 1997 60 4 928 934 9106540 PMC1712464

[B6] Veltman MW Thompson RJ Craig EE Dennis NR Roberts SE Moore V Brown JA Bolton PF A paternally inherited duplication in the Prader-Willi/Angelman syndrome critical region: a case and family study J Autism Dev Disord 2005 35 1 117 127 10.1007/s10803-004-1039-1 15796127

[B7] Mohandas TK Park JP Spellman RA Filiano JJ Mamourian AC Hawk AB Belloni DR Noll WW Moeschler JB Paternally derived de novo interstitial duplication of proximal 15q in a patient with developmental delay Am J Med Genet 1999 82 4 294 300 10.1002/(SICI)1096-8628(19990212)82:4<294::AID-AJMG4>3.0.CO;2-U 10051161

[B8] Gijsbers AC Bijlsma EK Weiss MM Bakker E Breuning MH Hoffer MJ Ruivenkamp CA A 400 kb duplication, 2.4 Mb triplication and 130 kb duplication of 9q34.3 in a patient with severe mental retardation Eur J Med Genet 2008 51 5 479 487 10.1016/j.ejmg.2008.04.003 18547887

[B9] Hou JW Wang TR Molecular cytogenetic studies of duplication 9q32-->q34.3 inserted into 9q13 Clin Genet 1995 48 3 148 150 8556822 10.1111/j.1399-0004.1995.tb04075.x

[B10] Mattina T Pierluigi M Mazzone D Scardilli S Perfumo C Mollica F Double partial trisomy 9q34.1-->qter and 21pter-->q22.11: FISH and clinical findings J Med Genet 1997 34 11 945 948 10.1136/jmg.34.11.945 9391894 PMC1051128

[B11] Spinner NB Lucas JN Poggensee M Jacquette M Schneider A Duplication 9q34-->qter identified by chromosome painting Am J Med Genet 1993 45 5 609 613 10.1002/ajmg.1320450519 8456834

[B12] Thorndike RL Hagen EP Sattler JM Technical Manual, Stanford-Binet Intelligence Scale 1986 Chicago, IL: The Riverside Publishing Company

[B13] Semel E Wiig EM Secord W Clinical Evaluation of Language Fundamentals-Revised (CELF-R) 1987 San Antonio, TX: Psychological Corporation

[B14] Mundy P Kasari C Sigman M Ruskin E Nonverbal communication and early language acquisition in children with Down syndrome and in normally developing children J Speech Hear Res 1995 38 1 157 167 7537345 10.1044/jshr.3801.157

[B15] Sparrow SS Cicchetti DV Diagnostic uses of the Vineland Adaptive Behavior Scales J Pediatr Psychol 1985 10 2 215 225 10.1093/jpepsy/10.2.215 4020603

[B16] Lord C Rutter M Le Couteur A Autism Diagnostic Interview-Revised: a revised version of a diagnostic interview for caregivers of individuals with possible pervasive developmental disorders J Autism Dev Disord 1994 24 5 659 685 10.1007/BF02172145 7814313

[B17] Lord C Rutter M Goode S Heemsbergen J Jordan H Mawhood L Schopler E Autism diagnostic observation schedule: a standardized observation of communicative and social behavior J Autism Dev Disord 1989 19 2 185 212 10.1007/BF02211841 2745388

[B18] Wang NJ Liu D Parokonny AS Schanen NC High-resolution molecular characterization of 15q11-q13 rearrangements by array comparative genomic hybridization (array CGH) with detection of gene dosage Am J Hum Genet 2004 75 2 267 281 10.1086/422854 15197683 PMC1216061

[B19] Makoff AJ Flomen RH Detailed analysis of 15q11-q14 sequence corrects errors and gaps in the public access sequence to fully reveal large segmental duplications at breakpoints for Prader-Willi, Angelman and inv dup(15) syndromes Genome Biol 2007 8 6 R114 10.1186/gb-2007-8-6-r114 17573966 PMC2394762

[B20] Battaglia A Gurrieri F Bertini E Bellacosa A Pomponi MG Paravatou-Petsotas M Mazza S Neri G The inv dup(15) syndrome: a clinically recognizable syndrome with altered behavior, mental retardation, and epilepsy Neurology 1997 48 4 1081 1086 9109904 10.1212/wnl.48.4.1081

[B21] Allderdice PW Eales B Onyett H Sprague W Henderson K Lefeuvre PA Pal G Duplication 9q34 syndrome Am J Hum Genet 1983 35 5 1005 1019 6613995 PMC1685803

[B22] Yatsenko SA Brundage EK Roney EK Cheung SW Chinault AC Lupski JR Molecular mechanisms for subtelomeric rearrangements associated with the 9q34.3 microdeletion syndrome Hum Mol Genet 2009 18 11 1924 1936 10.1093/hmg/ddp114 19293338 PMC2678925

[B23] Yatsenko SA Cheung SW Scott DA Nowaczyk MJ Tarnopolsky M Naidu S Bibat G Patel A Leroy JG Scaglia F Deletion 9q34.3 syndrome: genotype-phenotype correlations and an extended deletion in a patient with features of Opitz C trigonocephaly J Med Genet 2005 42 4 328 335 10.1136/jmg.2004.028258 15805160 PMC1736036

[B24] Wang NJ Parokonny AS Thatcher KN Driscoll J Malone BM Dorrani N Sigman M Lasalle JM Schanen NC Multiple forms of atypical rearrangements generating supernumerary derivative chromosome 15 BMC Genet 2008 9 1 2 10.1186/1471-2156-9-2 18177502 PMC2249594

[B25] Kubota T Sutcliffe JS Aradhya S Gillessen-Kaesbach G Christian SL Horsthemke B Beaudet AL Ledbetter DH Validation studies of SNRPN methylation as a diagnostic test for Prader-Willi syndrome Am J Med Genet 1996 66 1 77 80 10.1002/(SICI)1096-8628(19961202)66:1<77::AID-AJMG18>3.0.CO;2-N 8957518

[B26] Kubota T Das S Christian SL Baylin SB Herman JG Ledbetter DH Methylation-specific PCR simplifies imprinting analysis Nat Genet 1997 16 1 16 17 9140389 10.1038/ng0597-15

